# Latitudinal Diversity Gradients in New World Bats: Are They a Consequence of Niche Conservatism?

**DOI:** 10.1371/journal.pone.0069245

**Published:** 2013-07-23

**Authors:** Maria João Ramos Pereira, Jorge M. Palmeirim

**Affiliations:** 1 Department of Biology and Centre for Environmental and Marine Studies, University of Aveiro, Aveiro, Portugal; 2 Centre for Environmental Biology, Department of Animal Biology, Faculty of Sciences of the University of Lisbon, Lisbon, Portugal; Università degli Studi di Napoli Federico II, Italy

## Abstract

The increase in species diversity from the Poles to the Equator is a major biogeographic pattern, but the mechanisms underlying it remain obscure. Our aim is to contribute to their clarification by describing the latitudinal gradients in species richness and in evolutionary age of species of New World bats, and testing if those patterns may be explained by the niche conservatism hypothesis. Maps of species ranges were used to estimate species richness in a 100 x 100 km grid. Root distances in a molecular phylogeny were used as a proxy for the age of species, and the mean root distance of the species in each cell of the grid was estimated. Generalised additive models were used to relate latitude with both species richness and mean root distance. This was done for each of the three most specious bat families and for all Chiroptera combined. Species richness increases towards the Equator in the whole of the Chiroptera and in the Phyllostomidae and Molossidae, families that radiated in the tropics, but the opposite trend is observed in the Vespertilionidae, which has a presumed temperate origin. In the whole of the Chiroptera, and in the three main families, there were more basal species in the higher latitudes, and more derived species in tropical areas. In general, our results were not consistent with the predictions of niche conservatism. Tropical niche conservatism seems to keep bat clades of tropical origin from colonizing temperate zones, as they lack adaptations to survive cold winters, such as the capacity to hibernate. However, the lower diversity of Vespertilionidae in the Neotropics is better explained by competition with a diverse pre-existing community of bats than by niche conservatism.

## Introduction

The dramatic increase in species diversity from the Poles to the Equator is one of the most obvious biogeographic patterns, but the mechanisms underlying it remain quite obscure. The relevance of contemporary ecological mechanisms, such as climate, to the development of this pattern is widely recognized, but since it developed over evolutionary time scales it may also be a consequence of historical factors. The relative importance of contemporary and historical factors in the formation of latitudinal patterns in species richness is currently an active area of research [1].

Niche conservatism is defined as the retention of niche-related ecological traits over time [2]. It has been hypothesised that niche conservatism is important to explain how ecology and climate act on evolutionary and biogeographic processes (e.g. speciation, dispersal, extinction, invasions) to determine large scale patterns of species richness [3–6]. This hypothesis is based on evidence that most of the components of the fundamental niche, which describes the set of abiotic conditions in which a species is able to persist [sensu 7], are conserved over long evolutionary time scales. Such conservatism may constrain the present geographic range of species [2,4], and consequently influence latitudinal biogeographic patterns.

The niche conservatism hypothesis has received increasing support from ecologists [2,8,9], though recent reviews by Pearman et al. [10] and Losos [11] suggested that the prevalence of niche conservatism may have been overstated. Analysing studies done during the last decade they found that niche conservatism seems to occur in some clades for some traits, but that niches may also exhibit great evolutionary lability. For this reason, more empirical studies that address the theoretical predictions of niche conservatism are needed.

The consequences of niche conservatism on species richness allow the formulation of testable predictions [2,4,12–14]. First, if species tend to be unable to persist outside the conditions of their original fundamental niche, then higher species richness is expected in regions with environmental conditions closer to those that characterized the ancestral niches of the clades. Second, if the ecological preferences of basal clades are closely linked to climatic conditions, then basal taxa should be mostly confined to regions with climatic conditions similar to those prevailing where the group originated; conversely, more derived taxa are expected in regions with dissimilar conditions, because they needed more time to evolve and adapt to those conditions. Third, if niche conservatism drives the latitudinal richness pattern, then this pattern should be mostly determined by the distribution of basal taxa. Consequently, total richness should show a stronger positive spatial correlation with the richness of basal taxa than with the richness of derived taxa. Frequently, this prediction is associated with the tropical niche conservatism hypothesis, according to which more clades will be adapted to tropical climates because of the larger area under tropical climate over time [15].

New World bats are a good group to test the niche conservatism hypothesis because they include over 300 species [16] of which 249 are present in South America [17], and at the ordinal level follow the typical latitudinal increase in species richness towards the tropics [18–22]. In addition, there is sound information on their radiation [23], thanks to recent studies based on molecular, morphological, and fossil data [24,25].

The overall objective of this paper is to contribute to the clarification of the potential role of niche conservatism in the establishment of latitudinal diversity trends in New World bats. To do this we (1) described the latitudinal gradients in both species richness and evolutionary age for New World bats as a whole, and separately for its three largest families, and (2) tested if the observed latitudinal diversity patterns, under the light of the known evolutionary history of the Chiroptera, are compatible with the predictions of the niche conservatism hypothesis. We based these tests on predictions analogous to those used by Hawkins et al. [13,14,26]. If niche conservatism is a dominant mechanism underlining the latitudinal diversity gradient in New World bats then more species and, in average, older (evolutionarily more basal) species should occur in areas that retain climatic conditions similar to those prevailing during the radiation of the bat clades. In addition, the spatial correlation between the total bat species richness and the species richness of basal taxa should be higher than that between the total species richness and the species richness of derived taxa. We tested these predictions not only for New World bats as a whole, but also separately for each of the three most speciose families - Phyllostomidae, Vespertilionidae and Molossidae.

## Methods

### Species range maps

We obtained maps of species ranges of New World bat species from NatureServe [27]. This dataset is the most comprehensive database of distribution ranges available, and at the time of this study included 305 of the New World bat species. We then rasterized these maps into a 100 x 100 km grid in ArcView 3.2 (ESRI Inc., Redlands, California, USA) and estimated species richness for all cells.

### Phylogenetic metrics

We assigned a root distance (RD) to each species, using the number of nodes separating the species from the base of a phylogenetic tree following the approach of Hawkins et al. [12–14,26,28]. We used RD as a proxy of the age of species: a higher number of nodes indicates a more derived taxon (‘clade rank’ in [29]). We obtained RD values from the mammal phylogenetic tree resolved to the species level presented by Bininda-Emonds et al. [30], which integrates the Jones et al. [31] pre-existing supertree for the Chiroptera. The divergence times throughout the Bininda-Emonds et al. [30] tree were estimated by a combination of fossil and/or molecular dates under the assumption of a local molecular clock or interpolated from empirically dated nodes using a pure birth model.

Using the RD values of all species present in each cell of the grid we then calculated a mean root distance (MRD) for each cell for the nine New World bat families, and for all the families combined. These node based MRD values are approximate measures the evolutionary development [29] of the local fauna in each cell of the grid. Even if RD contains no direct information on the ages of taxonomic groups, it is still a useful metric for phylogenies lacking branch lengths [32] because although evolutionary rates may have not been the same in all clades, higher numbers of nodes represent a younger species in the majority of radiation phenomena. In addition, even if there are a few clades that show equal number of nodes and have different ages, this problem was minimized by using the mean root distance of multiple species in each cell of the grid.

It was not possible to assign a RD to 56 of the 305 species in the NatureServe dataset because they were not included in the phylogenetic tree; we removed these species from analyses involving the age of the clades.

### Data analysis

To determine the direction and significance of the relationship between (1) species richness and latitude, and (2) MRD and latitude, we used generalised additive models (GAM), because they allow the inclusion of non-linear terms in the linear predictor term [33]. The models were calculated using the package mgcv implemented in R software [34]. Spatial autocorrelation may inflate estimates of statistical significance, so we tested for its presence using Moran’s *I* values obtained at 10 different distance classes to create correlograms of the residuals of the initial models, using SAM software [35]. The statistical significance of Moran’s *I* (*P* < 0.05) is based on distances by randomization (using a Monte Carlo procedure with 200 permutations).

To test the prediction that total species richness follows the richness pattern of basal taxa more closely than that of the derived taxa, we calculated Pearson correlation coefficients between total richness and both basal and derived richness. To estimate these two types of richness we first ranked all the species from most basal to most derived and then defined as basal and derived the species in the 25% and 75% quartiles, respectively. Richness was then calculated separately using the sets of species included in each of these quartiles. As basal and derived groups are constrained to be correlated to the total data set (from which they were extracted), the spatial structure of total, basal and derived data sets was examined using Moran’s I autocorrelation coefficients obtained at 10 distance classes. Moran’s I coefficients for basal and derived taxa were then correlated against the Moran’s I of all taxa to assess the similarity of the spatial pattern of each of the subgroups with the pattern of the whole of the taxa using a major-axis model II regression implemented in RMA (http://www.bio.sdsu.edu/pub/andy/RMA.html); slope, R^2^ and associated standard errors estimates were calculated using one-delete jacknife procedure.

## Results

### Latitudinal patterns of richness and mean root distance

**Figure 1:**
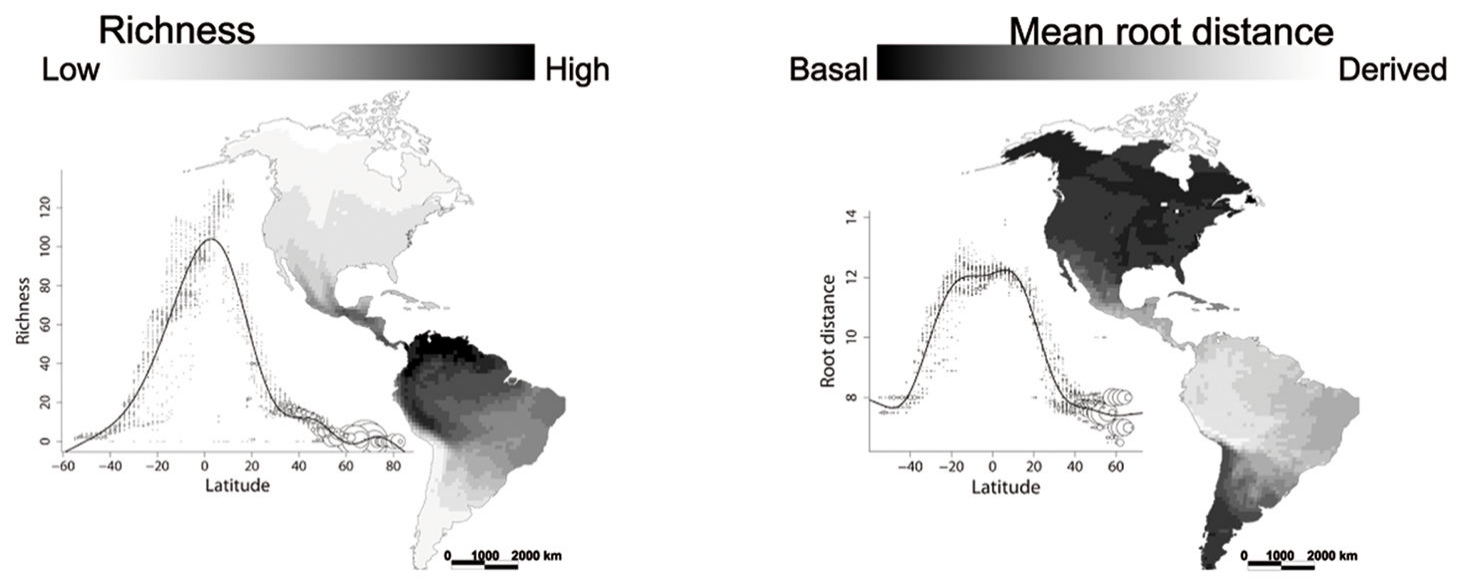
Geographical pattern of species richness (left) and mean root distance (right) in the Chiroptera resolved at a 100 x 100 km cell size. The plots show the relationships between latitude and species richness and between latitude and root distance. The size of the circles is proportional to the number of cells with the same bat richness at the corresponding latitude. The line shows the adjustment of the GAM model. White areas on the map indicate the absence of bats. All maps shown in Hammer-Aitoff equal-area projection.

**Figure 2:**
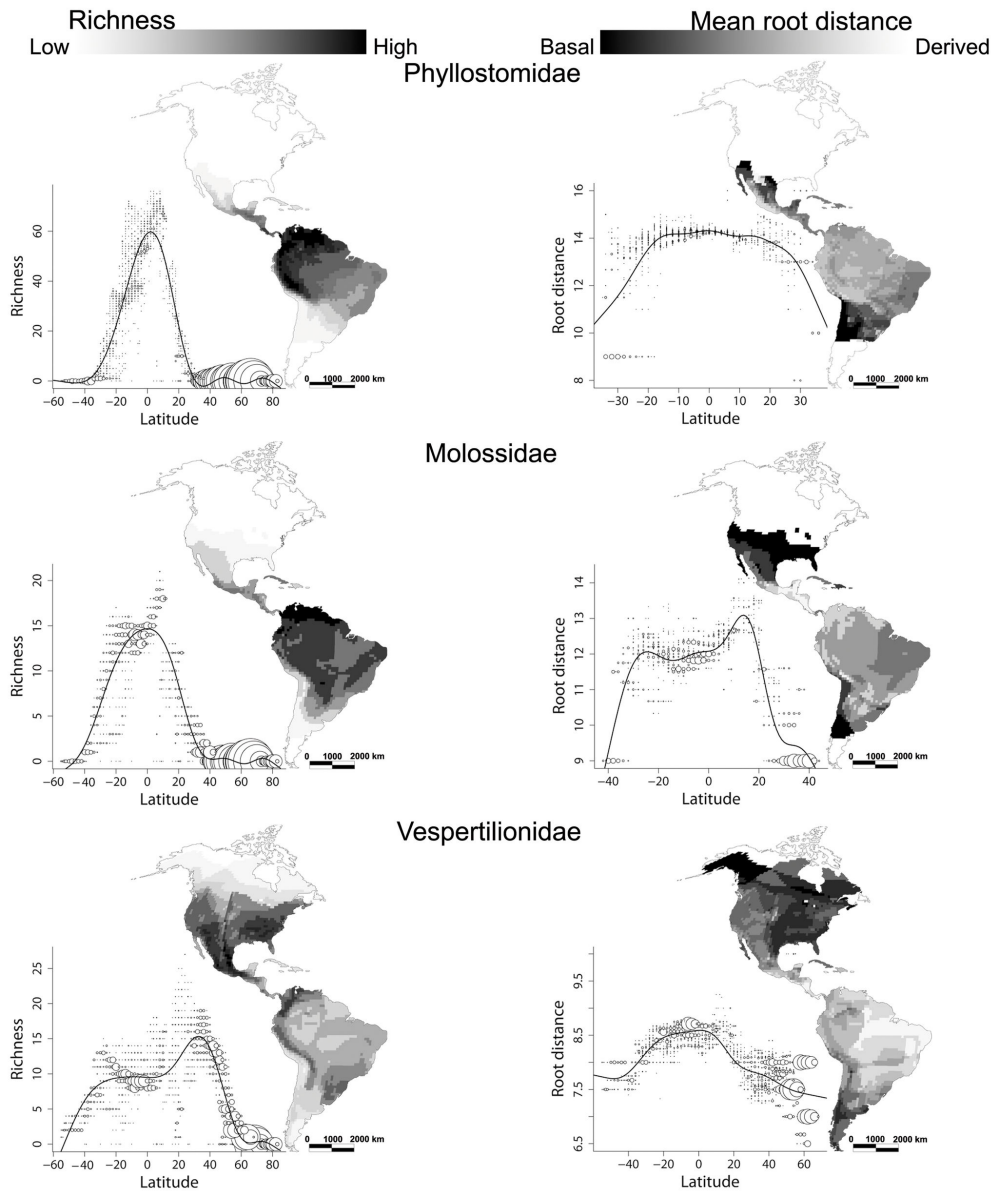
Geographical pattern of species richness (left) and mean root distance (right) in the Phyllostomidae, Molossidae, and Vespertilionidae resolved at a 100 x 100 km cell size, and relationships between latitude and species richness and between latitude and root distance using GAM. The size of the circles is proportional to the number of cells with the same bat richness at the corresponding latitude. The line shows the adjustment of the GAM model. White areas on the map indicate the absence of bats. All models were significant at p < 0.001.

Both species richness and MRD were closely correlated with latitude in the Chiroptera (Figure 1). Species richness is greater in tropical regions and more evolutionary basal species are found in higher latitudes. The percentage of deviance explained by the GAM was of 91.0% for species richness and 89.8% for MRD. Both models were significant at p < 0.001. With the exception of the Vespertilionidae (Figure 2) all the families showed a richness latitudinal pattern similar to the general pattern of the Chiroptera, with an increase of the number of species towards the tropical regions. However, the number of species in some families is too limited to generate robust spatial patterns in species richness and MRD, so we only present separate analyses for the three most speciose families (Figure 2).

The Phyllostomidae are absent from high latitudes and their species richness tends to increase towards the Equator, reaching a clear peak just north of it (Figure 2). The percentage of deviance explained by the GAM was 90.6%. Within the geographic range of the family more basal species tend to occur at higher latitudes, towards the temperate zones, but there is no discernable latitudinal trend within the tropical zones. The variation in MRD explained by the GAM was 43.9%. The latitudinal patterns observed in the Molossidae are very similar to those of the Phyllostomidae. Their species richness also peaks just north of the Equator and declines with latitude, but they penetrate further into the temperate zones. MRD is greater in the tropics: more basal species are found at the highest latitudes within the distributional range of the family, but the MRD remains relatively stable across tropical latitudes. The percentage of deviance explained by latitude was 94.3% and 79.2% in the richness and MRD models, respectively. The Vespertilionidae is the family with the broadest latitudinal range, and is present at almost all latitudes. The species richness pattern contrasts with those of the other families, as the peak of richness is not in the tropics but in temperate North America (Figure 2). In South America the regions of greater diversity are located in temperate or montane areas. The percentage of deviance explained by the GAM was 80.7%. Like in other bat families MRD increases towards the tropics, with more basal species present at the highest latitudes. Although the density of basal vespertilionid species is higher in the Northern Hemisphere, they also occur in temperate areas of South America. The deviance explained by latitude on the root distance GAM was 32.7%.

**Figure 3:**
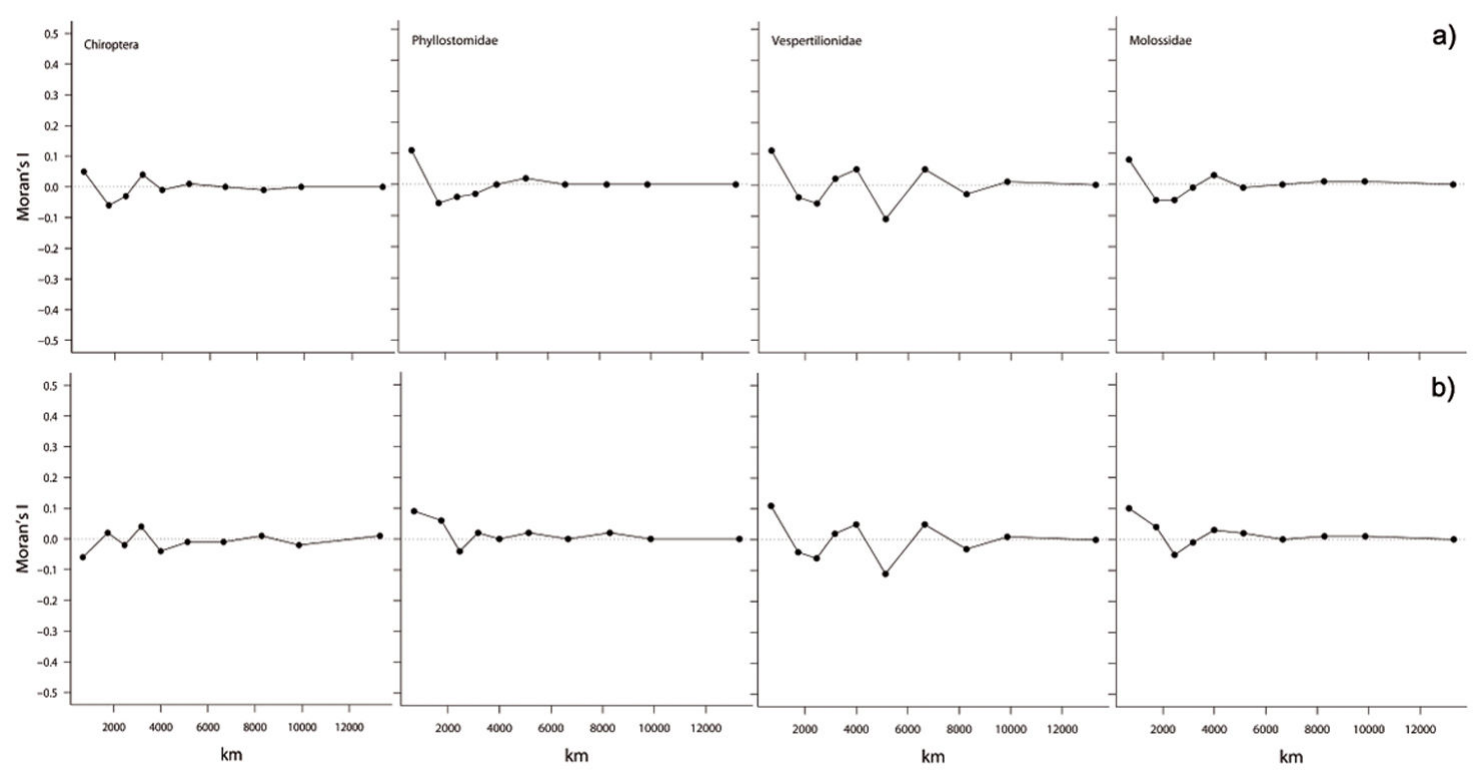
Moran’s *I* correlograms of residuals after fitting the models for species richness (top) and mean root distance (bottom). None of the values is significant at α = 0.05.

Moran’s *I* values did not show significant spatial autocorrelation even at the finest scale of analysis, for both the richness and root distance GAM (Figure 3). Indeed, even in the models with lowest percentage of deviance explained by latitude, Moran’s *I* values rarely exceeded 0.1. For this reason, there was no need to subsample cells to generate spatially independent data sets to adjust the final GAM.

Using data from bat assemblages of 30 New World sites, Stevens [36] reported a slight decline in root distance towards higher latitudes. To investigate if this apparent conflict with our results was due to the use of distinct phylogenetic trees or different distributional data, we applied MRD measured on the tree [37] used by Stevens [36] to our dataset of distributional ranges. The results (not shown) were very similar to those we had obtained with the Bininda-Emonds et al. [30] supertree.

### Relationship between total, basal, and derived species richness

**Figure 4:**
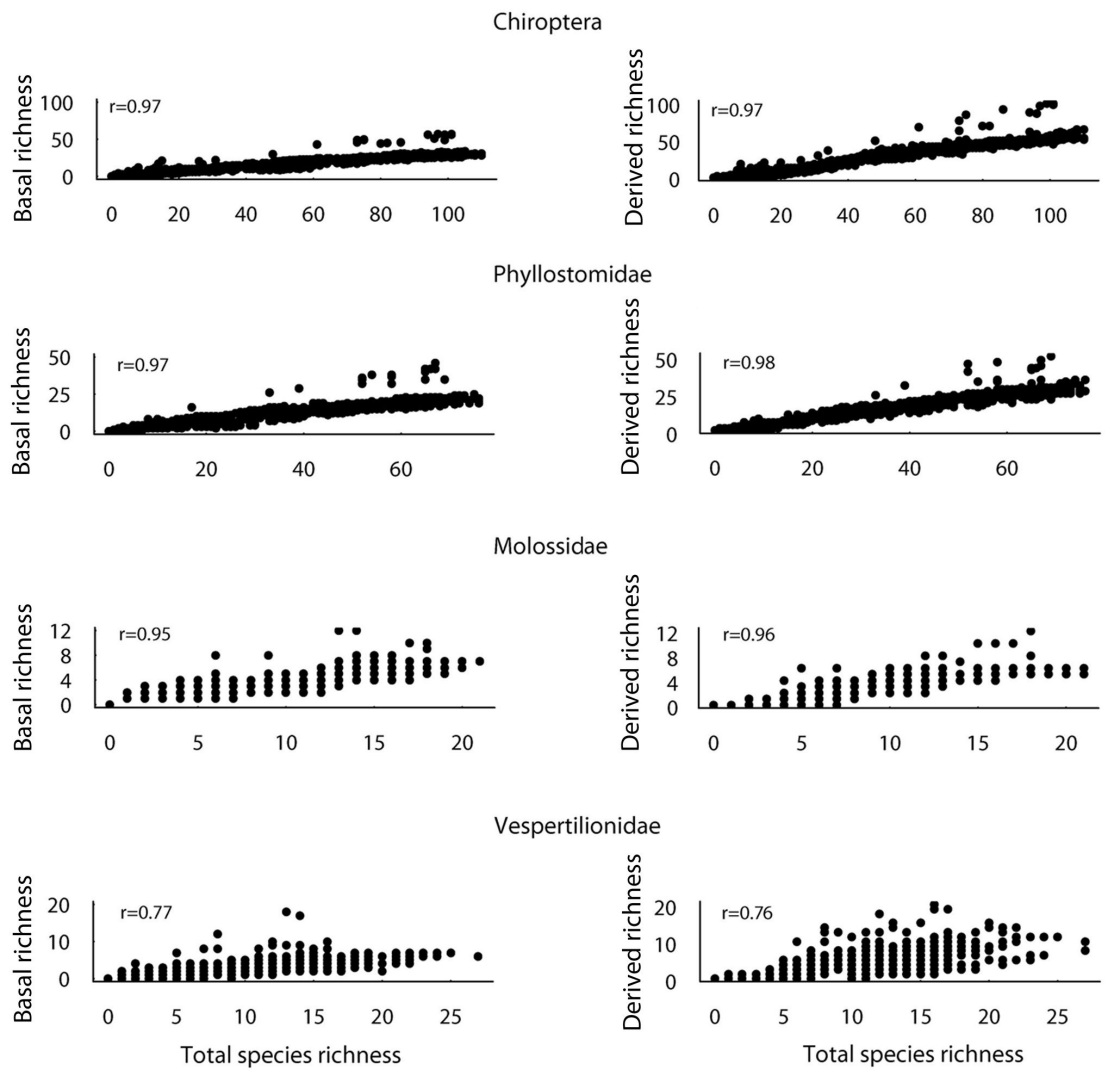
Relationships between total species richness and the richness of basal and derived bats for all species pooled and separately for the families Phyllostomidae, Molossidae and Vespertilionidae. Pearson correlation coefficient (r) between total richness and basal and derived richness is shown above each figure.

**Figure 5:**
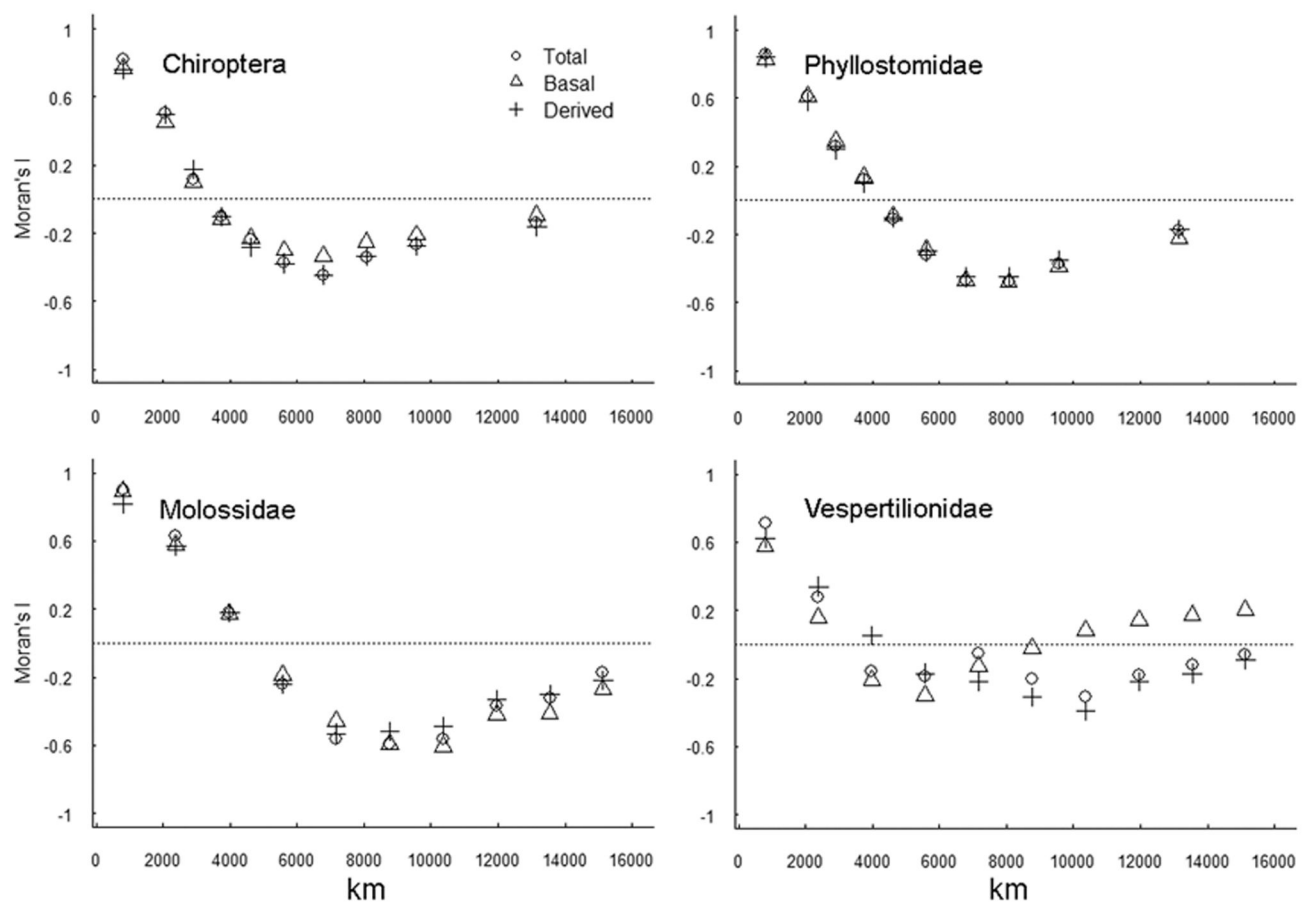
Spatial correlograms for total species and for basal and derived species in the Chiroptera and in the families Phyllostomidae, Molossidae and Vespertilionidae.

In the whole of the Chiroptera and separately in the Phyllostomidae, Molossidae and Vespertilionidae, both the basal and the derived species richness are strongly correlated with the overall species richness (Figure 4). The spatial correlogram for total species richness shows a strong positive autocorrelation at distances <2000 km in the order and in all families (Figure 5). The patterns for basal and derived species are very similar to the general pattern in the Phyllostomidae and Molossidae. Type II major-axis regression of the Moran’s *I* values of the total richness against the *I*s of basal and derived richness resulted in slopes approximately equal to 1 in the Phyllostomidae (total *vs* basal: b = 1.01 ± 0.02, *R* ^2^ = 0.99; total *vs* derived: b = 1.04 ± 0.01, *R* ^2^ = 0.99) and Molossidae (total *vs* basal: b = 1.00 ± 0.03, *R* ^2^ = 0.99; total *vs* derived: b = 1.09 ± 0.01, *R* ^2^ = 0.99). Only in the Vespertilionidae there was a slight deviance of the Moran *I*s of the total richness against the *I*s of basal richness (total *vs* basal: b = 1.33 ± 0.36, *R* ^2^ = 0.67; total *vs* derived: b = 1.07 +-0.19, *R* ^2^ = 0.83).

## Discussion

### General patterns of richness and mean root distance

All bat super-families appear to have originated 55 to 50 Mya, following the Paleocene-Eocene Thermal Maximum [24,38], when most of the world climate was tropical. Niche conservatism would then predict greater bat richness in the tropics, because most clades originated and had more time to speciate under a tropical environment. This was indeed observed in our analysis with all New World bats pooled (Figure 1). The increase of bat richness towards the New World tropics has been interpreted as a consequence of contemporary environmental factors correlated with latitude [20,21,39].

A second prediction of niche conservatism, assuming a tropical origin for most bat families, is that more basal clades should be found in the tropics, while derived taxa that have gained adaptations to survive colder climates should be more prevalent in temperate regions. This prediction was not upheld in our analysis, as the highest latitudes in average harbour more basal bat clades than the tropics (Figures 1 and 2). This result contrasts with that reported for New World birds [13], which are represented by more derived clades at higher latitudes. The difference may be due to the fact that some ancient and highly speciose bird families, such as the *Tina* *midae* , Cracidae, and Psittacidae, are restricted or nearly restricted to the tropics, whereas in bats most of the families of Neotropical origin are quite derived. This is the case of the Phyllostomidae, a comparatively derived clade that is very influential in the analysis because it includes many species.

The third prediction of the niche conservatism hypothesis, a greater spatial correlation between the total species richness and the species richness of basal taxa, was also not supported by our data. Indeed this correlation was similar to that between total species richness and the richness of derived taxa.

Our failure to find support for these predictions suggests that the overall increase of bat diversity towards the Equator should not be interpreted as a simple consequence of niche conservatism. However, this failure could be partly a consequence of a heterogeneous origin for the families of bats included in these analyses. This is so because the predictions are relative to the origin of the clades; if the various families of Chiroptera have different climatic origins then their individual trends may differ, potentially cancelling out and hiding overall patterns. In fact, even if all the super-families of bats did originate in a tropical environment, the various families may have later radiated under different climatic conditions. In particular, the Vespertilionidae may have radiated in the temperate zone, as suggested by the earliest known occurrences of this lineage [24] and the molecular phylogeny of some of its genera (e.g. *Myotis* [40]).

To avoid this potential confounding effect we also analysed the predictions of niche conservatism separately for the three most speciose families. In both the Phyllostomidae and Molossidae, species richness increases towards the Equator, and more basal clades are found at the highest latitudes of their range. The Vespertilionidae show a different pattern, exhibiting a greater richness and more basal species in temperate regions. Their richness declines at the highest latitudes in both hemispheres because of the harsh climate, but the species that reach them tend to be basal.

### Does ‘tropical’ niche conservatism explain the latitudinal diversity patterns in the *Phyllostomidae* and *Molossidae*?

The Phyllostomidae apparently radiated in the warm and wet Middle Eocene South America [24], and reach their peak of richness just north of the Equator (Figure 2). There is virtually no latitudinal trend in MRD within the tropics but, contrarily to the predictions of niche conservatism, the few species reaching temperate zones tend to be on average more basal than the ones in the tropics (Figure 2). When applying the MRD measure from the tree [37] used by Stevens [36] to our distribution data we obtained equivalent results to those obtained using the Bininda-Emonds et al. [30] supertree. This suggests that the disagreement with the slight decline in root distance towards higher latitudes found by Stevens [36] is due to the differences in the distributional data, presumably mostly because his sites range from 21.1^°^ N to 24.1^°^ S, whereas our distribution maps reach all the way to the northern and southern limits of the family (37^°^ N and 35^°^ S, respectively).

The latitudinal trends in richness and MRD in the molossids parallel those of the phyllostomids (Figure 2) suggesting that the factors that determine them are similar for both. The analyses of the spatial correlation between total, basal, and derived richness also did not yield results consistent with the predictions of niche conservatism. Consequently, with the exception of an increase in diversity towards their tropical origin, none of the predictions was upheld for two of the largest New World tropical bat families. So can we rule out a role of tropical niche conservatism in the formation of the latitudinal richness trend in these families? As Wiens & Donoghue [4] point out, strong evidence for a role of niche conservatism can come from finding the eco-physiological traits that underlie the limits of the ranges of the clades, and that trait is quite evident: the lack of adaptations to cold winters. Without the ability to hibernate, these bats cannot survive the cold and food scarcity of temperate zone winters [41]. Therefore, and in spite of the contradictory results of the testing of the predictions, it can be said that the northern and southern limits of the two families are indeed a consequence of tropical niche conservatism.

It seems likely that the phyllostomids and molossids once occupied a broader latitudinal band, and that their present distribution is relictual, reflecting a contraction of their range to regions where warmer climates persist, as suggested by Hawkins et al. [13,14] for the ‘tropical’ clades of New World birds. In fact, during the warm Early Eocene, tropical and other thermophilic vegetation extended into higher latitudes [42], and this may have allowed the expansion of tropical bat families up to the mid-latitudes of North America. Climate cooling towards the Late Eocene [43], presumably caused the latitudinal retreat of clades specialised in thermophilic environments, such as the phyllostomids and molossids, because they lacked the plasticity to adapt to the new ecological and climatic conditions. Still, at least one Old World molossid, *Tadarida* *teniotis* , has the capacity to enter lethargy during the winter [44], ranging into the temperate zone. This shows some potential of molossids to adapt to cold winters, but their radiation in the temperate zones may have been constrained by other factors, such as competition with the very diverse insectivorous vespertilionids, which have true hibernation capacities.

It is worth noting in our results the clear decline in richness of phyllostomids and molossids with latitude within the tropical region, not accompanied by a corresponding decline in MRD. This suggests that niche conservatism is not a major determinant of this intra-tropical richness trend, which may instead be caused by ecological factors. There are other important macrogeographic trends, such as the east–west richness decline in South America, presumably due to factors like topography and rainfall, which are known to influence bat species richness [21]. . Islands are known to promote isolation and accelerate speciation processes [45–49] but this is not evident in the MRD of the Caribbean islands, which is quite similar to that of continental regions at the same latitude.

### Does ‘temperate’ niche conservatism explain the latitudinal diversity patterns in the *Vespertilionidae*?

The geographical origin of vespertilionids is still equivocal, but the earliest occurrences of the family [24] suggest a temperate zone radiation, possibly in the sequence of the conquest of hibernation. Niche conservatism hypothesis would predict higher species richness and more basal clades in temperate areas than in the tropics, and indeed both predictions are consistent with the observed trends (Figure 2). This would suggest that having radiated under a temperate climate the basal vespertilionids would have a reduced ability to colonize tropical environmental conditions, because some element of the fundamental niche had limited their expansion towards the Equator; only the most derived clades would have evolved sufficiently to break the barriers of this fundamental niche and expanded to the tropics. However, we find that this simple scenario is not very likely because although the number of vespertilionid species is greater in the temperate zones, a substantial proportion of the genus of this family is present the Neotropics. The argument is further weakened by the success of this family in the Old World tropics. Finally, it is important to note that vespertilionid clades are very diverse in Northern Central America with tropical and subtropical climates. This ability to colonize the Neotropics is also marked in birds and may be an important contributor to the latitudinal diversity gradient [50].

**Figure 6:**
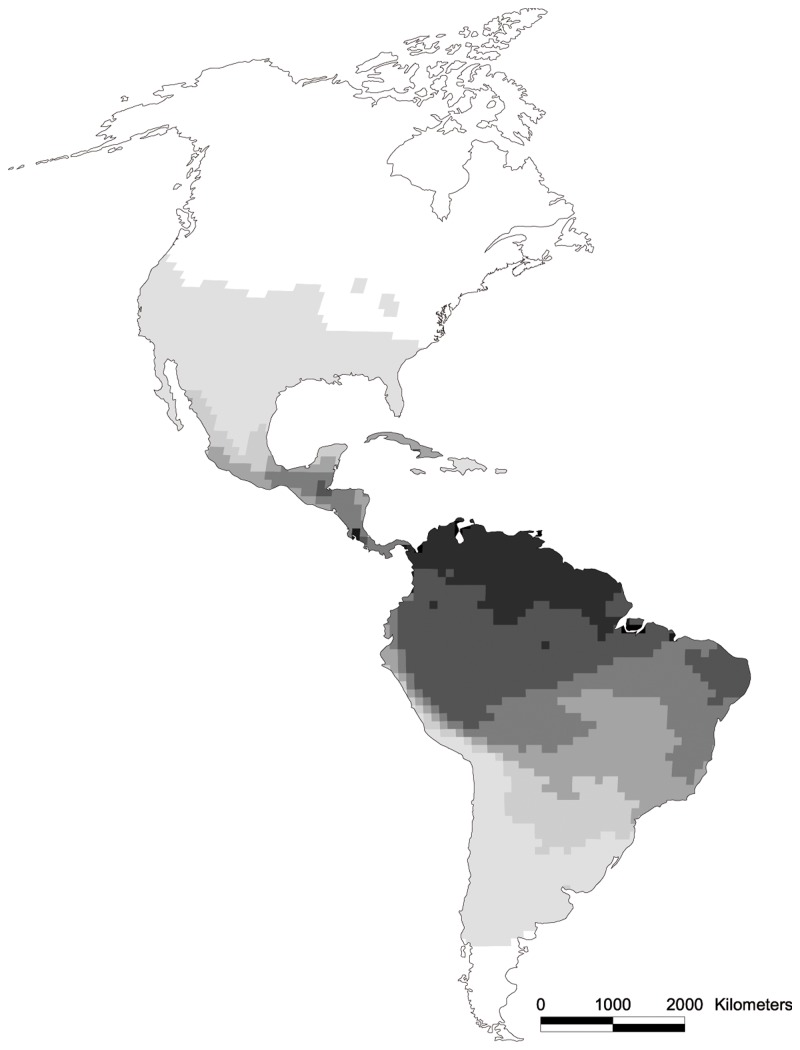
Geographical pattern of species richness in insectivorous bats belonging to the families Emballonuridae, Furipteridae, Molossidae, Mormoopidae, Phyllostomidae, and Thyropteridae resolved at a 100 x 100 km cell size. White areas indicate the absence of bats of those families.

This evidence argues against any intrinsic difficulty of the vespertilionids to adapt to Neotropical climatic conditions, and against a dominant role of niche conservatism to explain the decrease of species richness of this family towards the tropics. Then how can this departure from the typical latitudinal be explained? The Neotropics harbour the richest existing bat fauna, including many insectivores of the tropical families Emballonuridae, Furipteridae, Mormoopidae, Thyropteridae and Phyllostomidae. Vespertilionids presumably had a Laurasian origin [24] and, when they dispersed into South America met a diversified fauna of insectivorous bats, which evolved over a long period to fill the available niches. Competition from these bat families may have kept vespertilionids from diversifying in the Neotropics. This scenario of “late arrival” is compatible with the known molecular phylogenies of New World vespertilionids, such as that of *Myotis* [40],. Indeed *Myotis* presumably arrived to South America only 7-10 Mya [40] thus well after the appearance of the above referred Neotropical families [24], and of the arrival of the Emballonuridae to South America [51]. This “competition hypothesis” has been suggested before to explain the decrease in richness towards the Equator of *Myotis* [40,52]. It is consistent with the fact that the greatest richness of vespertilionids in intertropical South America is in montane regions, where the colder climate may reduce the competitive advantage of tropical bat clades (Figure 2). In addition, the richness of vespertilionids is almost the opposite of that of the other New World insectivorous bats taken together (Figure 6). So, the available evidence supports that the constraint to vespertilionid radiation in the Neotropics results from competition with other pre-established clades.

### How to explain the increasing proportion of derived bat species towards the Equator, regardless of their climatic origin?

The MRD increased from the temperate to the tropical region in all studied bat families, suggesting that niche conservatism is not the major determinant for the relative representation of basal and derived bat clades at different latitudes. It has been suggested that the greater prevalence of derived taxa of certain groups of organisms in the tropical regions may be a consequence of a tendency for faster rates of evolution in the tropics [53,54], although this theory is not supported by all analyses [55]; higher tropical temperatures may lead to faster individual growth rates and shorter generation times which could accelerate molecular evolution [56]. Indeed, the speed of growth of embryos and young bats can increase with temperature [57,58] and, whereas Holarctic bats are monoestrous, many tropical species are polyestrous [59]. Continual asynchronous breeding is common in Neotropical Phyllostomidae and Molossidae and even in some Neotropical vespertilionids such as *Myotis* *nigricans* [60]. These differences in the life cycle of temperate and tropical bats lead to shorter generation times in the latter potentially resulting in an increase in the rates of evolution and diversification.

An alternative explanation for the greater prevalence of derived bats in the tropics could be the environmental complexity hypothesis [61], according to which this complexity increases towards the tropics. So, as older species occupied the comparatively few niches available in temperate regions it became more difficult for new species to succeed. In the ecologically more diverse tropics, more niches could allow a greater success in speciation.

The increase in the proportion of basal species at higher latitudes in families of bats with a tropical origin could also be a consequence of a greater difficulty of successfully speciating in regions with cold winters. Without the ability to hibernate, additional successful adaptations are quite unlikely to occur, so speciation may actually be less frequent than in the tropical zones.

## Conclusions

In general, the predictions of niche conservatism related to the latitudinal distribution of derived and basal clades were not supported by our results, but this does not entirely rule out a role of niche conservatism to explain latitudinal richness trends in New World bats. Our analysis revealed that the situation is quite different for bats with a temperate origin (vespertilionids) and tropical origin (phyllostomids, molossids, and others).

Bat families with a tropical origin are virtually absent from the temperate zones because their evolution in a tropical environment did not prepare them for coping with cold winters, so tropical niche conservatism does play a role in the decrease of bat richness with latitude. However, competition with vespertilionids, which are well adapted to winter conditions, may also contribute to the near absence of tropical families in the temperate zones.

The only bat family with a presumed temperate origin, the vespertilionids, declines in diversity towards the Equator, contrasting with the general trend in the order. However, there is no evidence of any element of the fundamental (abiotic) niche of the family that would constrain its diversification under tropical conditions, so niche conservatism may not play an important role in the observed trend. Instead, the available evidence suggests that competition from the very diverse bat fauna that already existed in South America before their arrival may have limited vespertilionid richness in the region.

The mechanisms explaining the latitudinal richness patterns of New World bats are probably not the same for families with temperate and tropical origins. This suggests that in analyses done at the level of the order there is a risk that the patterns are confounded by different, and even opposing, tendencies. Although we tried to use the best possible information available, our conclusions may suffer from incomplete data on phylogenies and distributions data, and from uncertainties regarding the time and place of dispersal of bat clades. However, the results suggest that even if the predictions of niche conservatism about the latitudinal distribution of basal and derived clades are theoretically sound, they are not suitable to test for a role of niche conservatism in the development of latitudinal richness patterns in bats. This limitation may apply to other groups of organisms, so the testing of the theoretical predictions of niche conservatism must be addressed with caution.
